# Magnetism of materials: theory and practice in magnetic resonance imaging

**DOI:** 10.1186/s13244-021-01125-z

**Published:** 2021-12-04

**Authors:** Michele Gaeta, Marco Cavallaro, Sergio Lucio Vinci, Enricomaria Mormina, Alfredo Blandino, Maria Adele Marino, Francesca Granata, Agostino Tessitore, Karol Galletta, Tommaso D’Angelo, Carmela Visalli

**Affiliations:** Department of Biomedical Sciences and Morphological and Functional Imaging, Policlinico Universitario G. Martino, University of Messina, Via Consolare Valeria 1, 98100 Messina, Italy

**Keywords:** Magnetic resonance imaging (MRI), Magnetic susceptibility, Chemical shift, Artifact reduction

## Abstract

All substances exert magnetic properties in some extent when placed in an external magnetic field. Magnetic susceptibility represents a measure of the magnitude of magnetization of a certain substance when the external magnetic field is applied. Depending on the tendency to be repelled or attracted by the magnetic field and in the latter case on the magnitude of this effect, materials can be classified as diamagnetic or paramagnetic, superparamagnetic and ferromagnetic, respectively. Knowledge of type and extent of susceptibility of common endogenous and exogenous substances and how their magnetic properties affect the conventional sequences used in magnetic resonance imaging (MRI) can help recognize them and exalt or minimize their presence in the acquired images, so as to improve diagnosis in a wide variety of benign and malignant diseases. Furthermore, in the context of diamagnetic susceptibility, chemical shift imaging enables to assess the intra-voxel ratio between water and fat content, analyzing the tissue composition of various organs and allowing a precise fat quantification. The following article reviews the fundamental physical principles of magnetic susceptibility and examines the magnetic properties of the principal endogenous and exogenous substances of interest in MRI, providing potential through representative cases for improved diagnosis in daily clinical routine.

## Key points

- Every endogenous and exogenous substance is influenced by an applied magnetic field.
- Magnetic susceptibility permits to evaluate the magnitude of magnetization of materials.
- Knowledge of materials’ magnetic properties helps practitioners exploit at best MRI sequences.

## Introduction

Magnetic resonance imaging (MRI) is the medical application of nuclear magnetic resonance (NMR) phenomenon discovered and described simultaneously and independently in January 1946 by two teams led by Felix Bloch at Stanford and Edward Mills Purcell at the Massachusetts Institute of Technology, respectively. For their discovery, Bloch and Purcell were awarded with the Nobel Prize in Physics in 1952.

The idea of MRI was the brainchild of Dr. Raymond Damadian, who prefigured the possibility of using NMR to discriminate between malignant tumors and normal tissue in 1971 [1]. Paul Lauterbur and Peter Mansfield further developed MRI, and in 2003 they both received the Nobel Prize in Physiology or Medicine [2].

MRI provides information on anatomy and physiological processes using strong magnetic fields, magnetic field gradients, and radio waves to generate images of the body. More precisely, but in a very simple way, MRI can be defined as a grayscale map of the content of hydrogen protons and of their interactions with the surrounding atomic and molecular tissue environment.

In this review, we examine both theoretically and practically the effects of magnetic properties, and specifically of magnetic susceptibility, of human tissues and various endogenous and exogenous substances on MRI. We discuss the implication of these properties on daily MRI practice so as to obtain, exploiting at best MR sequences, high-quality images, thus potentially improving diagnosis and patients’ healthcare.

A complete description of the physical basis of MRI is beyond the scope of this article and has been extensively reviewed in numerous papers. However, some basic concepts of MRI physics will be briefly retrieved for the purposes of discussion.

## MRI physics

The normal image, in which the voxel grayscale reflects the magnitude of the MRI signal, is the magnitude image.

The main sources of MRI contrast in the human body are:

1. proton density (PD), which is the concentration or density of hydrogen protons in the tissues and is the most direct tissue characteristic that can be imaged;
2. longitudinal relaxation time (T1);
3. transverse relaxation time (T2).

PD, T1 and, T2 are intrinsic properties of matter when it is set in a magnetic field and interacts with electromagnetic waves of relevant energy [3–7].

**Fig. 1:**
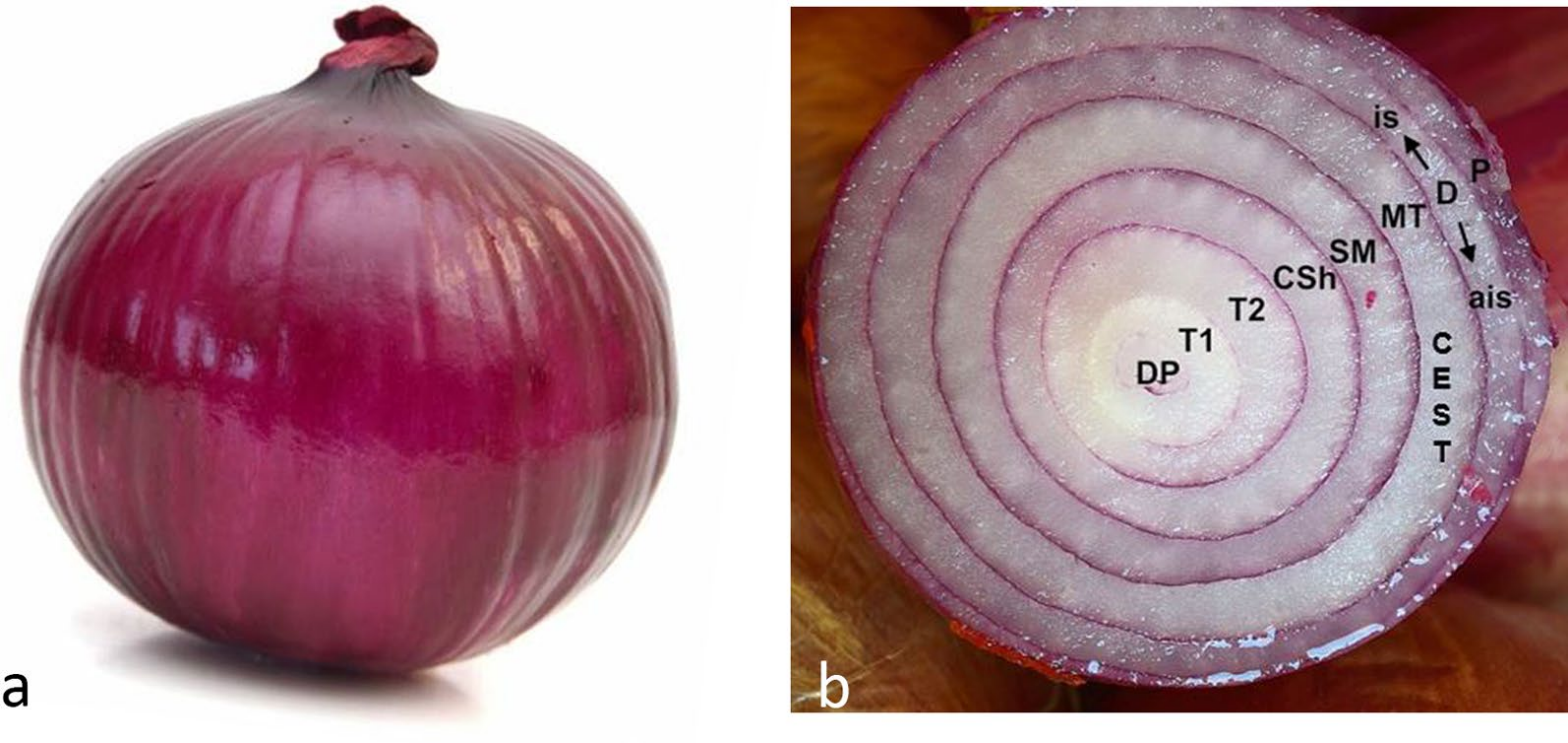
The magnitude image **a** is the sum of different intravoxel sources of MRI contrast (**b**), here schematically represented in an onion model. DP = Proton density; T1 = longitudinal relaxation time; T2 = transverse relaxation time; CSh = chemical shift; SM = magnetic susceptibility; MT = magnetization transfer; CEST = chemical exchange saturation transfer; D = diffusion, isotropic (is) and anisotropic (ais); P = Perfusion

There are other sources of MRI contrast, such as diffusion and perfusion, but they are beyond the scope of this paper [8, 9]. The sources of contrast in MRI are summarized in Fig. 1.

### Longitudinal relaxation time (T1)

When the human body is placed in an external magnetic field (*B*_0_), a nuclear magnetic vector (the net magnetization vector), which represents the averaged sum of the magnetic properties of all the singular nuclei within the body, develops. The application of radiofrequency (RF) waves, transmitted as brief bursts (RF-pulses), excites the nuclei, thus turning the net magnetization vector to a higher energy state. T1 relaxation represents the mechanism by which the net magnetization vector returns to its initial state in thermodynamic equilibrium with the surrounding environment. This mechanism is characterized by a time constant known as T1. More precisely, T1 is defined as the time required for the longitudinal component of the magnetization vector (longitudinal magnetization) to recover about 63% of its initial value (*B*_0_).

For the aims of this paper, it is important to recall that T1 relaxation is mainly a thermodynamic phenomenon: at the cessation of the radiofrequency impulse, the excited protons return to their previous status by transferring energy to the surrounding atoms and molecules in the form of heat (T1 relaxation is also known as spin–lattice relaxation, where lattice indicates the external environment). The more the molecular tumbling rate (that is, the thermodynamic movement of the atoms and molecules of the environment) is similar to the proton precession frequency, the faster the transfer of heat takes place. In the human body, at a temperature of 37° the molecular tumbling is usually faster than proton precession [10]. Thus, T1 shortening can be caused by two different mechanisms:

1. Slowing of the particles of the environment. Both an elevated concentration of high-weight molecules (e.g., molecules of fat, proteins and glycidic macromolecules, like glycoproteins of mucus) and cooling slow the molecular tumbling rate, thus shortening T1. Tissue cooling should always be considered in post-mortem examinations because it strongly influences MRI signal [11–14];
2. Fastening of protons. Paramagnetic substances (e.g., gadolinium) in the environment increase the proton precession frequency and consequently cause shortening of T1.

### Transverse relaxation time (T2)

Simultaneously to the above-mentioned processes, after the initial RF-pulse, the net magnetization vector is tilted to the transverse plane, due to “phase coherence” of nuclei (in fact, of only a minute fraction of nuclei) in the transverse plane. T2 relaxation, also known as spin–spin relaxation or transverse relaxation, refers to the progressive dephasing of these nuclei, resulting in decay of the net magnetization in the transverse plane. This form of relaxation occurs with the time constant T2, which is defined as the time required for the transverse component of the net magnetization vector to descend to approximately 37% of its initial value [10].

The major causes of dephasing of the transverse magnetization are:

1. presence of tiny magnetic fields of ~ 1 mT created by the spinning nuclei;
2. influence of the local (i.e., intra-tissue) magnetic field (*B*_*loc*_), characteristic of each type of molecule, which can affect spin phasing. Physiological molecules (e.g., water and fat) present differences in diamagnetic susceptibility that cause intra-voxel inhomogeneity. Deposition of molecules due to pathological conditions can also cause further changes of *B*_loc_.
3. simultaneous T1 relaxation. If the in-phase nuclei exchange energy with the surrounding environment, both the longitudinal and transverse components of the net magnetization vector will be affected, thus simultaneously influencing T1 and T2 relaxation (the so-called “T1 contribution to T2” or “T1 in T2”).
4. Dipolar interactions, consisting in exchange of longitudinal angular momentum between a pair of spins (also known as spin–spin “flip–flop” exchanges).

Some concepts about T2 relaxation are mandatory to remember:

1. The MRI electrical signal (the only source for MR images creation) is only generated by the movement of the transverse magnetization vector. Thus, reduction of net transverse magnetization due to T2 dephasing is accompanied by a proportional exponential decay of MRI signal. The exponential curve of MR signal decay is named “free induction decay (FID)”.
2. Spin echo (SE) and, much more, turbo spin echo (TSE) sequences reduce intra-tissue inhomogeneity using a 180° pulse that re-phase the transverse magnetization vector. On the other hand, sequences that do not use 180° impulses [namely, all gradient echo (GE) and echo-planar imaging (EPI) sequences] cause a shortening of T2, named T2* relaxation.
3. Variations of echo time (TE), by sampling FID at different points, strongly influence the effect of local inhomogeneity on MRI signal, both in T1- and T2-weighted (T1-w. and T2-w.) images. Namely, a short TE reduces, while long TEs enhance, the effects of *B*_loc_ inhomogeneity, respectively. The choice of different TEs can be used in practical MRI to reduce or increase the effect of local susceptibility differences in order to improve the diagnostic effectiveness.

## Types of magnetism

All substances, including human tissues, exhibit some form of magnetism, which is the distortion that a substance immersed in a magnetic field causes on the magnetic field itself.

In electromagnetism, the magnetic susceptibility (symbolized by the Greek letter *χ*) is a quantitative measure indicating the magnitude of magnetization of a material when an external magnetic field is applied [15].

The magnetic susceptibility (*χ*) of a material, which is a dimensionless quantity, is defined by the following equation:

1 $$\chi = \frac{M}{H}$$

where *M* is the magnetization of the material (quantity of magnetic moment per unit volume), and *H* is the magnetic field strength, both measured in ampere (A)/meter (m). In MRI, a strictly connected term, magnetizability, is used, which is the ratio of magnetization to magnetic flux density (i.e., the strength of the applied magnetic field *B*_0_, measured in Tesla (T)) [16]. For the purposes of this review article, susceptibility and magnetizability will be considered equivalent terms.

Four fundamental types of interaction between magnetic fields and matter exist, which define the magnetic characteristics of substances, dividing them into two groups: on the one hand, diamagnetic substances, which have negative susceptibility (*χ* < 0) and tend to be repelled by the magnetic field; on the other hand, paramagnetic, superparamagnetic and ferromagnetic substances, which have positive susceptibility (*χ* > 0) and tend to be attracted by the magnetic field. Specifically, we consider:

1. Diamagnetism: where *χ* is slightly negative, in the order of 10^–6^;
2. Paramagnetism: where *χ* is positive and typically in the range of 10^–5^–10^–3^. The magnitude of this susceptibility is less than 0.1% of that of ferromagnetic materials;
3. Superparamagnetism: where magnetic susceptibility is much larger than the one of paramagnets, but lower compared to ferromagnetic materials;
4. Ferromagnetism: where *χ* is positive and extremely large, typically greater than 100.

The main diamagnetic, paramagnetic, superparamagnetic and ferromagnetic substances of interest in MRI are listed in Table 1. It is important to recall that electron clouds (not nuclei) of atoms and molecules primarily determine magnetic susceptibility of a material. Atomic or molecular orbitals containing paired electrons contribute to diamagnetism; orbitals with unpaired electrons contribute to para-, super- and ferro-magnetism.

**Table 1:** Principal endogenous and exogenous substances, listed according to their magnetic susceptibility

**Diamagnetic**		
Water	Fat	Proteins
Calcium (Ca)	Collagen	Lead (Pb)
Oxyhemoglobin		
**Paramagnetic**		
Gadolinium (Gd)	Cupric ion (Cu^2+^)	Aluminum (Al)
Hydroxyl radical (·OH)	Magnesium (Mg)	Manganese (Mn)
Melanin	Methemoglobin	Ferrous Oxide (FeO)
Molecular oxygen (O_2_)	Titanium (Ti)	
**Superparamagnetic**		
Ferritin	Hemosiderin	Deoxyhemoglobin
**Ferromagnetic**		
Iron (Fe)	Cobalt (Co)	Nickel (Ni)
Alloys of iron (e.g., steel)	Alloys of rare-earth metals	

Other types of magnetism exist, such as ferrimagnetism, antiferromagnetism and metamagnetism, but are of no interest in MRI, and consequently not further considered in this review.

We are now going to discuss the magnetic properties of the primary endogenous and exogenous substances of interest in MRI and how their magnetism influences MR signal, providing numerous examples and focusing on clinical aspects, especially covering less known or less reported applications.

Subsequently, we will examine the principal MRI sequences that can be used to exalt or reduce these phenomena depending on the clinical context, including some practical issues to help radiologists optimize their use and avoid potential errors in interpretation.

## Magnetism of materials in clinical practice

### Imaging of diamagnetism and chemical shift

Diamagnetism is caused by the orbital motion of electrons creating tiny current loops, which produce weak magnetic fields. Almost all biological tissues are weakly diamagnetic, with calcium salts (present for instance in cortical bone) being the strongest diamagnetic substances in the human body. It is worth mentioning that air is not diamagnetic, because molecular oxygen (O_2_) is paramagnetic.

Diamagnetic differences between nuclei and molecules can be evaluated by spectroscopy, which is beyond the scope of this paper [17–19]. The most useful diamagnetic phenomenon exploited in daily MRI practice is represented by chemical shift imaging. Diamagnetic susceptibility phenomena at an atomic level constitute the physical basis of chemical shift. Indeed, hydrogen protons do not precess at exactly the same frequency, as they are influenced by the molecule to which they belong. The electron shell around the nucleus produces a shielding effect that opposes *B*_0_, so that every proton is subjected to a local magnetic field (*B*_loc_) different from *B*_0_. As a matter of fact, *B*_loc_*s* and consequently precession frequencies may be slightly different between two protons depending on the molecule in which they dwell. This difference of precession frequency is called chemical shift.

The most important chemical shift effect for MRI of the human body is due to differences in diamagnetism between water and fat. Unlike triglycerides, the water molecule is polarized with the electron cloud shifted toward oxygen, leaving hydrogen protons more exposed to *B*_0_. Thus, water and fat protons precess at slightly different rates with an average chemical shift difference of about 3.5 parts per million: this means, considering the external magnetic field strength, that water protons precession frequency is higher than fat protons precession rate of around 220 Hz at 1.5 T and 440 Hz at 3 T [20, 21]. Chemical shift imaging is obtained with in-phase/opposed-phase imaging and Dixon techniques, which permit to demonstrate the coexistence of water and fat within the same voxel [22, 23].

#### Clinical applications

**Fig. 2:**
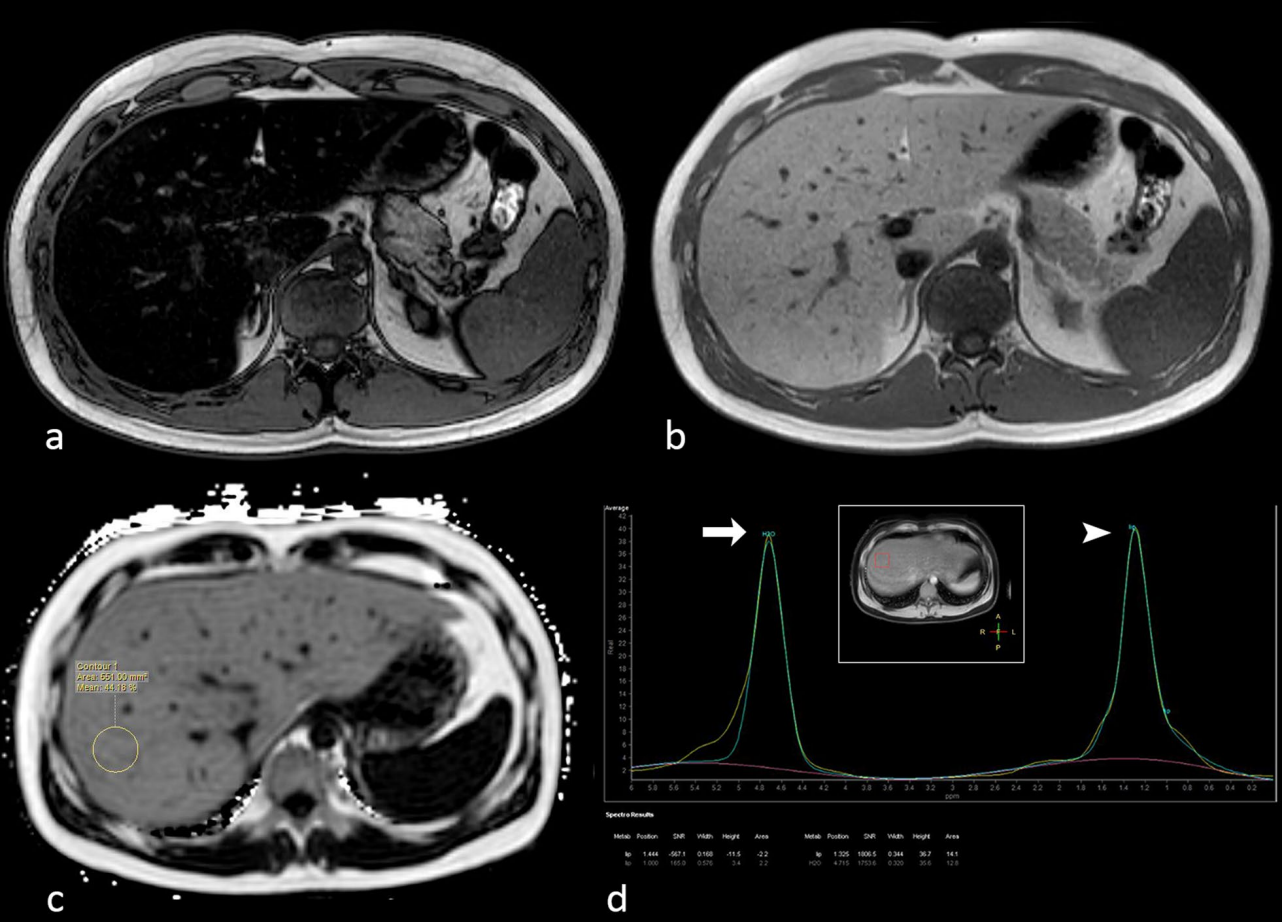
Opposed-phase (**a**) and in-phase (**b**) gradient echo (GE) T1-w. images of the upper abdomen show severe hepatic steatosis. A proton density fat fraction (PDFF) sequence (**c**) demonstrates a fat fraction of 44% in liver segment VIII. Hydrogen-spectroscopy (**d**) confirms the fat fraction quantification
  A further interesting application of fat quantification is in the field of muscular diseases. Since dead muscular cells are replaced by intramuscular fat, fat amount correlates with loss of muscle and consequently with severity of disease [29–34].
- Bone marrow evaluation
  Chemical shift imaging can also be exploited for studying bone marrow. Since red bone marrow contains 40% of water and 40% of fat, it can be explored with dual GE T1-w. sequence; this is particularly useful for detection of diseases in which red marrow is completely substituted, mainly in primary and secondary bone cancer [35]. In normal conditions, red bone marrow shows signal dropout on out-of-phase images. Thus, pathologic tissues, which substitute red marrow and do not contain fat, can be easily detected (Fig. 3). In addition, in-phase and out-of-phase images are useful to demonstrate bone marrow reconversion that can simulate bone disease [36–39].

- Fat detection and quantification
  One of the most common and studied application of chemical shift imaging is fat detection in liver. Dual (in-phase and opposed-phase) GE T1-w. sequence permits to identify hepatic steatosis, by detecting signal intensity loss on the out-of-phase image compared with the in-phase image. The signal dropout can be seen when the fat fraction is more than 10–15% and is maximal when the magnitude of fat signal is identical to that of water signal, which corresponds to a 50% fatty infiltration. If the fat fraction is above this threshold, however, the signal intensity of out-of-phase image starts to increase and water and fat cancellation is not optimal [21].
  Nowadays, specific sequences, which represent an evolution of the standard dual GE sequence, can be used to obtain fat-quantification [24–27]. These GE sequences use multiple TEs (multi-echo GE sequences) and low flip angles. A low flip angle reduces T1-effects, while multi-echo acquisition permits correction of T2* effects. Fat fraction can then be calculated as a ratio from fat and water signal.
  Fat quantification is now a mandatory technique for evaluating patients with hepatic steatosis since it allows an evaluation of fat-fraction comparable to that of hepatic biopsy, in a fast, non-invasive and repeatable way [28] (Fig. 2).

**Fig. 3:**
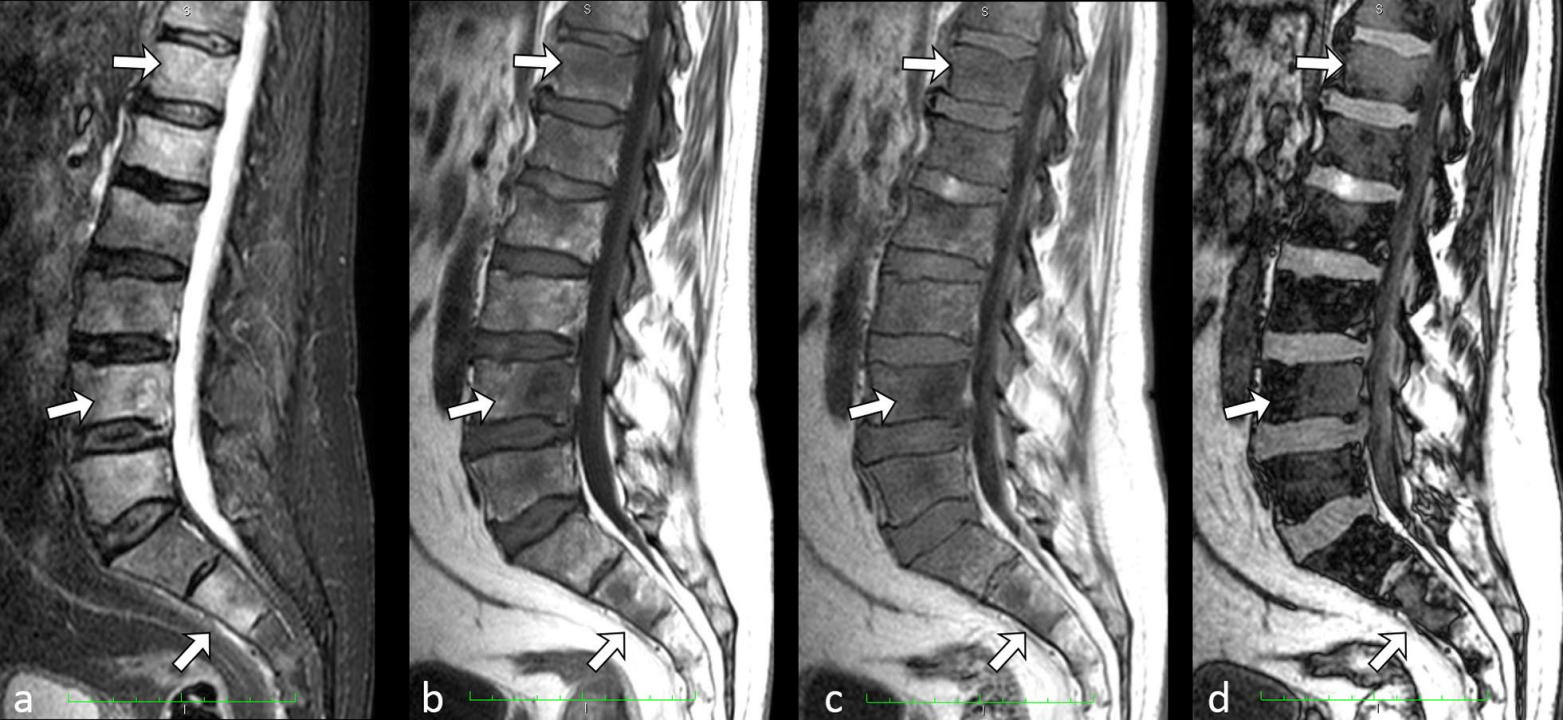
Sagittal short inversion time inversion recovery (STIR) (**a**) and T1-w. turbo spin echo (TSE) (**b**) series showing multiple, ill-defined, lumbar vertebral body metastatic lesions in a 57-year-old patient with known lung cancer. Sagittal in-phase (**c**) and opposed-phase (**d**) gradient echo (GE) T1-w. series in the same patient. Opposed-phase image shows with great advantage and better details the various lesions, as areas of red bone marrow substitution. Arrows indicate the most prominent lesions
- Other applications
  Chemical shift imaging is routinely performed to diagnose common pathologic conditions, and its use is largely discussed in the international literature. The main applications include the diagnosis of adrenal adenomas, thymic hyperplasia, chylothorax; furthermore, it can help distinguish hepatocellular carcinoma (HCC) and hepatocellular adenoma with intralesional fat from different lesions in the liver [21, 40–42].

### Imaging of paramagnetism

Every paramagnetic material presents unpaired electrons in the atomic or molecular orbitals. When an external magnetic field is applied, the unpaired electrons are free to align their magnetic moment in the same direction of the applied field, thus reinforcing it.

The human body contains some paramagnetic substances, both in physiological and pathological conditions. When the intra-tissue concentration of these substances is high enough, they change the MRI signal so that they can be detected.

On T1-w. sequences the presence of paramagnetic substances generates high signal intensity. In addition, a high concentration of paramagnetic substances affects T2-w. images as well, causing loss of signal. This phenomenon is dependent on two mechanisms:

1. a strong paramagnetic intra-tissue field causes fast dephasing of transverse magnetization (shortening of T2) with consequent weakening of MR signal;
2. a marked shortening of T1 affects T2, due to the “T1 contribution to T2.” If T1 is shorter than the TE of the T2-w. sequence, the FID is completely faded off at the time of echo-sampling, so no signal can be measured.

The list of paramagnetic substances that can be found in human tissues includes methemoglobin (Met-Hb), melanin, hydroxyl radicals, gadolinium, manganese, cupric ion and molecular oxygen. Paramagnetic materials also include some metals, such as aluminum, titanium and ferrous oxide.

#### Gadolinium

Gadolinium generates a magnetic field a thousand times stronger than water protons and is the main contrast agent used in MRI. In addition, gadolinium can accumulate in brain and cause high signal intensity on T1 “unenhanced” images [43–46].

#### Hydroxyl radicals (·OH)

**Fig. 4:**
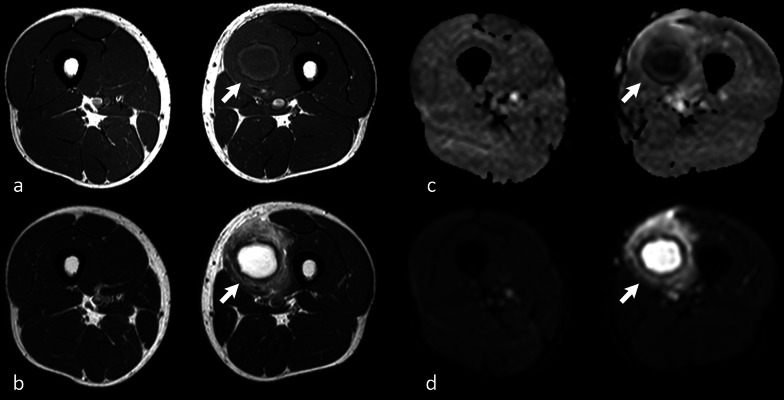
27-year-old male patient complaining of swelling and worsening pain in the left thigh for a week. Axial turbo spin echo (TSE) nonenhanced T1-w. image **a** shows a cystic round lesion (arrow) within the vastus medialis muscle of the left thigh. TSE T2-w. scan (**b**) at the same level well depicts the edema of the muscle around the lesion. A clear, subtle rim (hyperintense in **a** and strongly hypointense in **b**) encircling the cystic component can be noted, representing the lesion wall. The mass shows typical central restricted diffusion on the apparent diffusion coefficient map (**c**) and on the diffusion-weighted imaging (**d**) sequence. The findings were consistent with muscular abscess (arrows)

Hydroxyl radicals are found in macrophages. Abscesses generally present innumerable macrophages within their wall; thus, the abscess wall appears hyperintense on T1-w. images and dark on T2-w. images (Fig. 4).

#### Methemoglobin (met-Hb)

Methemoglobin (met-Hb) is the product of intra-tissue hemorrhage. Met-Hb molecule presents five unpaired electrons. In addition, its structure allows close approach of water molecules to the ferric ion (Fe^*3*+^) of the heme group. Consequently, met-Hb is highly paramagnetic and its presence results in very short T1 values.

In the setting of hemorrhagic events, during the first week met-Hb is concentrated into multiple intracellular compartments, generating local magnetic susceptibility effects, so that hematoma appears dark on T2/T2*-w. images. After the first week, lysis of red blood cells occurs with release and dispersion of met-Hb into the extracellular spaces. Consequently, local T2* dephasing effects disappear and the hematoma appears bright on T2-w. images [47].

#### Melanin

Melanin has a high affinity and binding capacity for metal ions and natural melanin contains a wide variety of bound metals (iron, copper, manganese and zinc) and hydroxyls in vivo. The T1 and T2 shortening of natural melanin reflects the paramagnetic effect of these bound paramagnetic substances [48, 49].

**Fig. 5:**
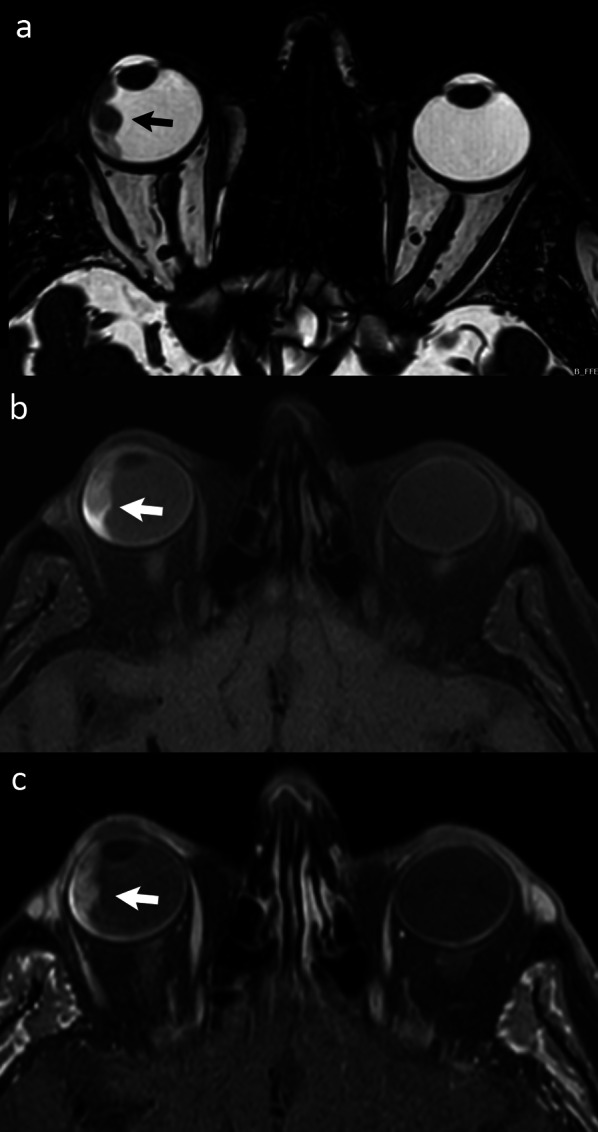
Axial balanced fast field echo (bFFE) MR image (**a**) showing an irregular and hypointense tissue plaque along the lateral wall of the right eye. Nonenhanced turbo spin echo (TSE) after spectral presaturation with inversion recovery (SPIR) T1-w. scan **b** demonstrates that the lesion is hyperintense. On contrast-enhanced TSE T1-w. image (obtained after subtraction of signal of nonenhanced image) (**c**) enhancement of the lesion can be seen, demonstrating that the lesion is vascularized. The findings are consistent with melanoma (arrows)

Melanin is normally contained in some brain nuclei as substantia nigra (neuromelanin). Melanin within primitive melanoma and its metastases causes hyperintensity on T1-w. images, which is a very useful finding that helps suspect the nature of the lesion [50, 51]. However, melanin might hide contrast-enhancement after gadolinium administration. Thus, it is mandatory in case of a lesion containing melanin to obtain subtraction images (T1-gadolinium minus T1-basal) in order to demonstrate the enhancement of the cancer (Fig. 5).

#### Molecular oxygen (O_2_)

**Fig. 6:**
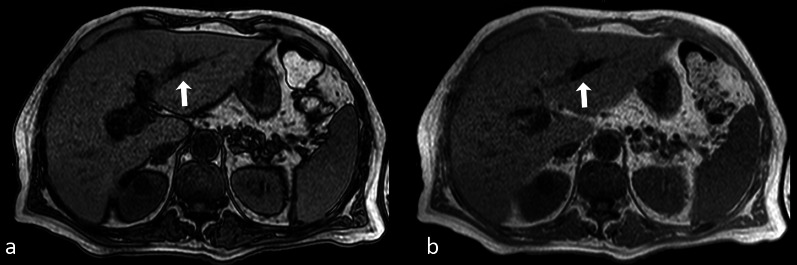
Axial opposed-phase (**a**) and in-phase (**b**) GE images showing a linear susceptibility-related artifact in the left hepatic duct, consistent with pneumobilia. The hypointensity is more conspicuous on the in-phase image

The presence of molecular oxygen in the air contributes to the susceptibility effects in every pathologic process containing gas, like abscesses or pneumobilia (Fig. 6).

#### Manganese (Mn)

In acquired hepatocerebral degeneration, a high pre-contrast signal intensity is often observed in the globus pallidus, in the subthalamic region and in midbrain (mainly in the substantia nigra), due to increased tissue concentrations of manganese within these structures [52–54].

#### Cupric ion (CU^2+^)

Copper (Cu) in its ground state is diamagnetic because it does not contain unpaired electrons. On the other hand, its ion Cu^2+^ contains unpaired electrons and is paramagnetic.

**Fig. 7:**
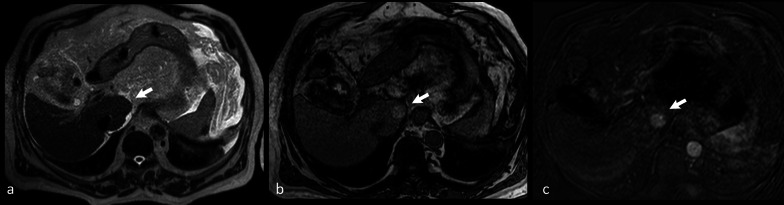
Axial turbo spin echo (TSE) T2-w. image **a** of the abdomen does not show any focal lesion on the caudate lobe of the liver (arrow). On nonenhanced opposed-phase gradient echo (GE) T1-w. image **b** a well-defined hyperintense nodule (arrow) at the same level can be identified. Contrast-enhanced fat-saturated GE T1-w. scan in the arterial phase (obtained after subtraction of signal of nonenhanced image) (**c**) shows distinct contrast-enhancement of the lesion (arrow). Final diagnosis was low-grade hepatocellular carcinoma

Cirrhotic nodules, including regenerative nodules, dysplastic nodules and HCC can appear hyperintense on unenhanced T1-w. imaging [55–57] (Fig. 7).

According to Chou et al., in cirrhotic liver 44% of T1-w. hyperintense nodules were dysplastic nodules, and the remaining 56% were nodules of HCC [58]. Thus, the mere hyperintensity on T1-w. images does not allow a differential diagnosis between benign and malignant hepatic nodules. Focal hepatic lesions showing hyperintensity on unenhanced T1-w. imaging might be due to lesions containing T1-shortening elements (e.g., glycogen, fat, copper, highly concentrated proteins) [58, 59]. Namely, concentration of paramagnetic Cu^2+^ ions has been demonstrated in hyperintense hepatic nodules [60].

#### Paramagnetic metals

**Fig. 8:**
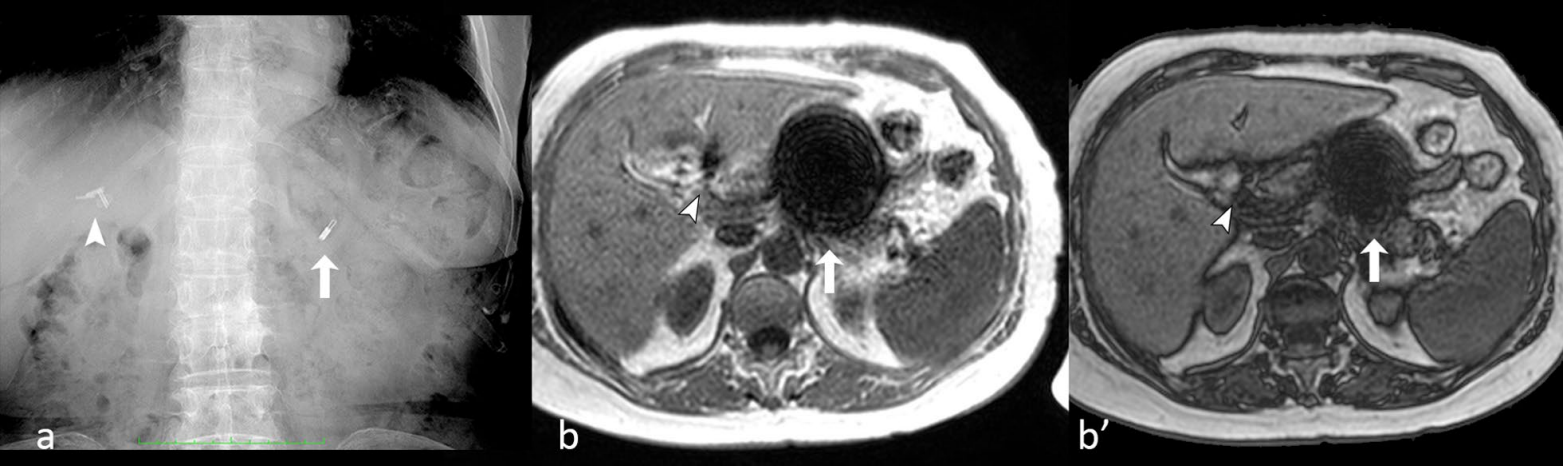
Abdominal X-ray (**a**) showing two metallic devices in the right (arrowhead) and left (arrow) upper quadrants, respectively. The devices cause magnetic susceptibility-related artifacts on in-phase (**b**) and opposed-phase (**b′**) gradient echo (GE) T1-w. images in the stomach (arrow) and hepatic hilum (arrowhead), respectively; the signal intensity loss is higher on in-phase image (**b**). It is worthy of note that the magnetic device in the stomach (a ferromagnetic steel clip) causes a much more prominent artifact than the device in hepatic hilum (a paramagnetic titanium clip by previous cholecystectomy)

It is important to know that several metals which are widely used in surgery, like titanium and aluminum, are paramagnetic. If more than one different metallic device is present in a scan, the magnitude of the susceptibility-related artifact can help characterize the metal as paramagnetic, superparamagnetic or ferromagnetic, thus diagnosing the different nature (Fig. 8).

### Imaging of superparamagnetism and ferromagnetism

Similarly to paramagnetic materials, superparamagnetic and ferromagnetic substances also have unpaired electrons, but generate a higher susceptibility effect.

In ferromagnetic materials, the magnetic moments of the electrons tend to align parallel to each other (in addition to the external magnetic field); therefore, their magnetism is higher compared to paramagnetic materials.

If a ferromagnet (or ferrimagnet) is sufficiently small and single-domain, it acts like a single magnetic spin that is subject to Brownian motion, creating a strong positive susceptibility effect known as superparamagnetism. Though stronger compared to the susceptibility generated by paramagnetic substances, the susceptibility of superparamagnetic materials is lower than the ferromagnetic one; furthermore, when the external magnetic field is removed, superparamagnetic materials do not keep a magnetization, differently from ferromagnetic substances.

Imaging of superparamagnetism and ferromagnetism is a matter of great importance in MRI.

Ferromagnetism occurs in a few substances; common examples include iron, nickel, cobalt, their alloys (e.g., steel) and some alloys of rare-earth metals. These substances are often contained in metallic prostheses used in surgery and can be found in the human body (Fig. 8).

On the other hand, deoxyhemoglobin in venous blood and some products of degradation of hemoglobin, namely ferritin and hemosiderin, are superparamagnetic.

#### Deoxyhemoglobin

Superparamagnetism of deoxyhemoglobin is exploited for functional MRI (fMRI) of the brain. fMRI measures the small changes in blood flow related to brain activity: the energy consumption of brain cells causes increases of blood flow and differences in the content of deoxyhemoglobin between active and inactive brain areas [61].

#### Ferritin and hemosiderin

Ferritin is made of a hollow proteic shell and a core storing up to 4500 iron atoms. Each iron atom in ferritin has three unpaired electrons. When placed in an external magnetic field, spins from these thousands of electrons lock together, generating a strong paramagnetic effect. The ferritin core can be considered as a solid piece of iron with a single magnetic domain.

On the other hand, hemosiderin is an iron-containing amorphous substance without a fixed composition. It is made up of conglomerates of clumped ferritin particles, proteins and lipids. Hemosiderin is stored in macrophages, and, in the brain, in glial cells.

Because its particles are larger than those of ferritin, magnetic susceptibility effects of hemosiderin are more powerful.

Both ferritin and hemosiderin give rise to marked T2 shortening, so that the areas where they accumulate appear dark on MR image [62–66].

**Fig. 9:**
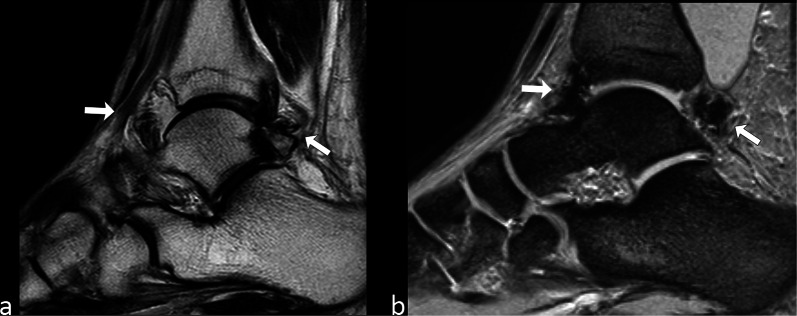
Images of the right ankle (**a** and **b**) in a 35-year-old man with hemophilia. Presence of intra-articular hemosiderin (arrows) is seen with greater advantage on GE T2*-w. image (**b**) compared to TSE T2-w. scan (**a**)

Presence of hemosiderin is common in giant cell tumor of the tendon sheaths and in intra-articular pigmented villonodular synovitis. Intra-articular hemosiderin can also be found in patients with hemophilia after recurrent hemorrhage (Fig. 9).

## MRI sequences and magnetic susceptibility

### Gradient echo (GE) T2*-w. and susceptibility weighted imaging (SWI) sequences

**Fig. 10:**
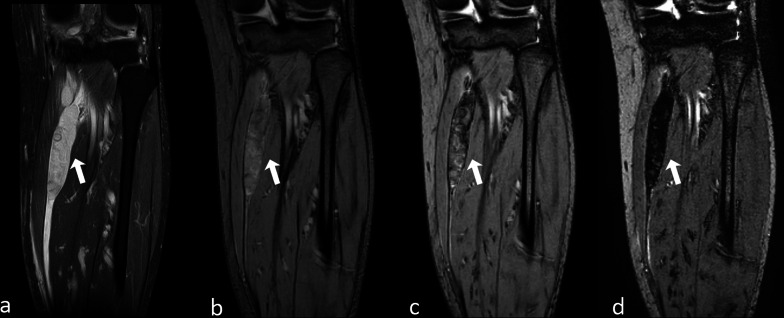
A 58-year-old woman with symptoms of chronic compartment syndrome of the right lower leg. Coronal turbo spin echo (TSE) T2-w. fat-saturated image **a** shows an ovoidal mass (arrow) between the muscles of the posterior compartment. Contrast-enhanced study (not shown) was inconclusive. On gradient echo (GE) T2*-w. images obtained simultaneously with different echoes—8, 16 and 40 ms (**b–d**), respectively—evident and homogeneous blooming of the entire lesion allows to characterize the mass as a hematoma (arrow)

GE T2*-w. sequence is a well-known optimal technique to demonstrate the presence of intra-voxel differences of susceptibility due to the presence of superparamagnetic, ferromagnetic or highly concentrated paramagnetic materials (Fig. 9). A useful related technique is the multi-echo GE T2*-w. sequence, which improves the visibility of various susceptibility sources and facilitates diagnostic interpretation. It allows to maximize artifacts (the so-called “blooming artifact”), a finding which is very specific and can help differentiate loss of signal due to susceptibility artifact from signal hypointensity due to different sources (e.g., fibrosis) [67]. Such an increase of blooming in multiple longer echoes has been named super-blooming and allows to detect even small amounts of strongly paramagnetic, superparamagnetic or ferromagnetic substances in tissues [68] (Fig. 10).

An evolution of GE T2*-w. sequences, which improves the detectability of susceptibility artifacts is susceptibility weighted imaging (SWI) [69–72]. Broadly speaking, SWI could be considered the evolution of blood oxygenation-level-dependent (BOLD) imaging (the standard technique used to generate images in fMRI studies), which exploits the differences of susceptibility between diamagnetic oxygenated hemoglobin (oxyHb) and superparamagnetic deoxygenated hemoglobin (deoxyHb) [61, 66, 73–75]. SWI combines T2*-w. magnitude image and phase image acquired with a GE sequence. SWI generates a better contrast than GE T2* sequence between tissues of differing susceptibility.

### In-phase/opposed-phase imaging

As formerly discussed, dual (in-phase and opposed-phase) GE T1-w. sequences allow to demonstrate the coexistence of water and fat within the same voxel and constitute the standard imaging technique for chemical shift imaging. However, in-phase/opposed-phase imaging can also be exploited to study conditions associated with signal intensity loss due to magnetic susceptibility phenomena.

**Fig. 11:**
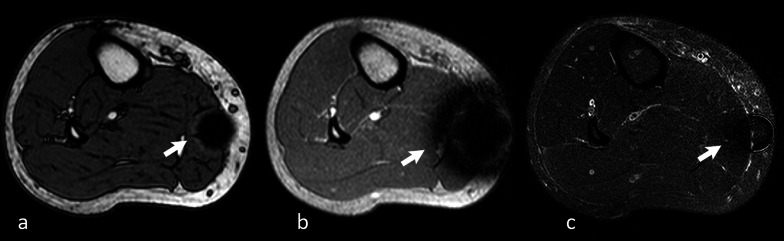
Axial opposed-phase (**a**) and in-phase (**b**) gradient echo (GE) T1-w. and short inversion time inversion recovery (STIR) **c** series of the left leg in a 35-year-old patient with a distinct susceptibility artifact due to a superficial iron surgical clip (arrow). A marked enlargement of the metallic artifact is noted in the in-phase image [echo time (TE): 4.6 ms] compared to the opposed-phase image (TE: 2.3 ms). On STIR sequence (TE: 40 ms) the artifact is less evident despite the use of a longer TE, due to the use of multiple re-phasing 180° pulses (turbo spin echo factor: 20)

**Fig. 12:**
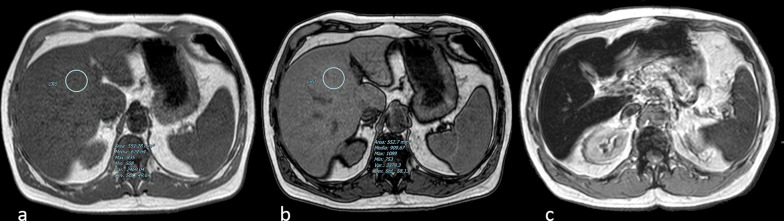
Magnetic susceptibility-related signal intensity loss on in-phase gradient echo (GE) T1-w. image **a** with respect to opposed-phase image (**b**). The signal intensity loss is around 30% and is due to presence of hemosiderin in a patient with hepatic hemosiderosis. On turbo spin echo (TSE) T2-w. image (**c**) a marked hypointensity of the liver confirms the diagnosis of intra-hepatic hemosiderin deposition

These sequences, lacking a 180° rephasing pulse, are more sensitive to susceptibility-induced intratissue inhomogeneities, compared to SE and TSE sequences. Therefore, the presence of substances with positive susceptibility causes a T2* decay-related signal intensity loss and, when in-phase and opposed-phase images are compared, this signal intensity loss is higher on the in-phase image, where the longer TE allows more chance for T2* decay to verify [21]. Thus, conditions associated with deposition of paramagnetic, superparamagnetic or ferromagnetic substances (like pneumobilia, liver hemochromatosis or hemosiderosis) or presence of metallic foreign bodies (such as surgical clips) can be detected and characterized on the in-phase image due to magnetic susceptibility-related signal intensity loss (Figs. 6, 8, 11, 12).

This effect has to be kept in mind when applying in-phase/opposed-phase imaging for “chemical shift” purposes, as it can lead to errors of interpretation. For instance, in a liver with excess iron due to hemochromatosis or in regions close to metallic surgical clips, the signal loss due to the T2*-effects can alter signal dropout due to fat–water cancellation and mask hepatic steatosis or interfere with fat quantification [76].

### Inversion recovery sequences

Inversion recovery (IR) sequences are strongly dependent on the T1 of tissues. More precisely, IR sequences can null the signal of certain tissues by the selection of an appropriate value of inversion time (TI), defined “null point” (TI_null_), according to the following equation:

2 $${\text{TI}}_{{{\text{null}}}} = T1 \times \ln 2 \approx T1 \times 0.69$$

#### Short TI inversion recovery (STIR)

Among the most important IR sequences, short TI inversion recovery (STIR) sequence is commonly used to suppress the signal from fat. At 1.5 Tesla the T1 of fat is approximately 250 ms, with a TI_null_ of 170 ms.

**Fig. 13:**
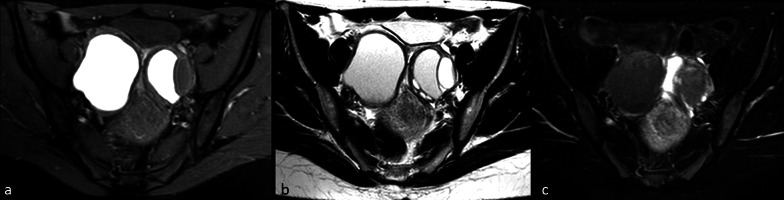
32-year-old woman with chronic pelvic pain. Axial T1-w. fat-saturated scan (**a**) of the pelvis shows two large hyperintense endometrial cysts. Axial T2-w. scan (**b**) through the same level demonstrates a slight “shading” phenomenon in the cysts. In the short inversion time inversion recovery (STIR) image (**c**) the signal of the cysts is cancelled because they have the same T1 of the fat

STIR usually ensures an effective fat suppression (using a TI of 170 ms), which is relatively resistant to magnetic field inhomogeneities. However, fat saturation with STIR is nonselective, given that other tissues with the same T1 of the fat are cancelled as well. Thus, paramagnetic substances, which—as we previously mentioned—tend to shorten T1, may determine signal suppression on STIR images. A common example of misdiagnosis using STIR sequences is represented by endometriomas, which can be wrongly diagnosed as fat-containing pelvic lesions. Shortening of T1 in endometrial cysts is a combination of high protein concentration (thermodynamic shortening) and accumulation of paramagnetic blood products as a result of chronic intra-cyst hemorrhage (electromagnetic shortening) (Fig. 13).

**Fig. 14:**
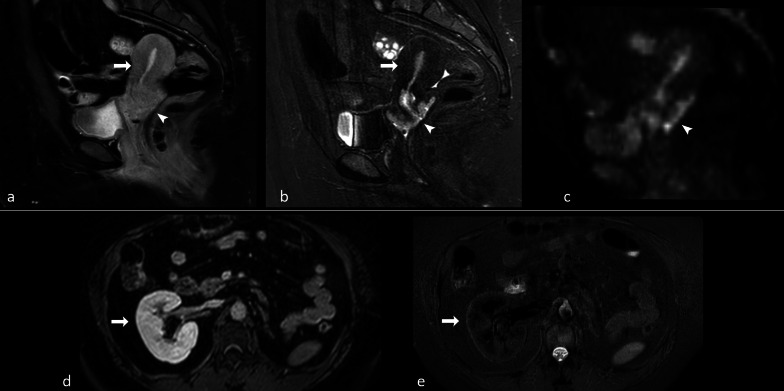
38-year-old patient with cervical carcinoma. On contrast-enhanced turbo spin echo (TSE) T1-w. fat-saturated sagittal image (**a**) differentiation between normal uterus and cancer is difficult. On sagittal short inversion time inversion recovery (STIR) scan (**b**) obtained at the same level immediately after TSE sequence, the signal of normal uterus is cancelled and hyperintense cancer is well depicted. There is an optimal correspondence between STIR and diffusion-weighted image (**c**), where spatial resolution is however lower. Contrast-enhanced gradient echo (GE) T1-w. fat-saturated scan (**d**) through the right kidney. Contrast-enhanced fast-STIR image (**e**) obtained immediately after d shows that the signal of renal parenchyma is cancelled since its T1 is equal to that of fat

In view of their non-selectivity, STIR sequences are generally not recommended to be used after administration of gadolinium for fat suppression, as both enhanced organs and lesions can show a loss of signal comparable to that of fat (e.g., normal renal parenchyma, Fig. 14d, e) and other sequences are usually preferred [77, 78]. However, this phenomenon could be exploited to better delineate a tumoral lesion in some specific cases. For instance, in case of uterine cancer the exact definition of tumor margins in relation to normal parenchyma is often challenging. It is possible that the usage of STIR sequence following gadolinium administration may lead to a better delineation of the lesion in comparison with conventional contrast-enhanced T1-w. fat-suppressed TSE images, as the correspondence between STIR and diffusion-weighted images seems to suggest (Fig. 14a–c). However, future studies are needed to better clarify these topics.

#### Fluid attenuated inversion recovery (FLAIR)

**Fig. 15:**
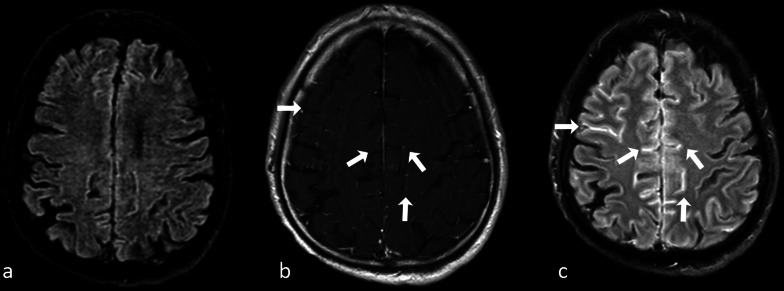
25-year-old female patient with suspected meningitis. Nonenhanced fluid attenuated inversion recovery (FLAIR) **a** image appears negative. Turbo spin echo (TSE) T1-w. magnetization transfer scan **b** is inconclusive, although mild effacement of some frontal and parietal sulci can be suspected (arrows). On contrast-enhanced FLAIR (**c**) scan extensive hyperintensity of several sulci can be easily diagnosed (arrows)

A different IR sequence which has recently been proved to be highly useful after gadolinium in the setting of meningitis is fluid attenuated inversion recovery (FLAIR) sequence [79]. Indeed, in patients with meningitis, FLAIR sequence acquired after gadolinium administration has been shown to be more effective than contrast-enhanced T1-w. images for depicting meningeal inflammation, since the amount of gadolinium passing into the cerebrospinal fluid (CSF) may not be enough to be perceived on T1 images, but can still be sufficient to change T1 values of CSF to the point that CSF is not adequately suppressed on FLAIR images (Fig. 15).

### Echo-planar imaging (EPI)

Echo-planar imaging (EPI) is performed using a pulse sequence in which multiple echoes of different phase steps are acquired using rephasing gradients. This is accomplished by rapidly reversing the readout or frequency-encoding gradient [80–83].

EPI is the fastest acquisition method in MRI (100 ms/slice), but with limited spatial resolution. It is based on:

- an excitation pulse, possibly preceded by magnetization preparation;
- continuous signal acquisition in the form of a gradient echo train, in order to acquire total or partial k-space (single shot or segmented acquisition);
- readout and phase-encoding gradients adapted to spatial image encoding, with several possible trajectories to fill k-space.

**Fig. 16:**
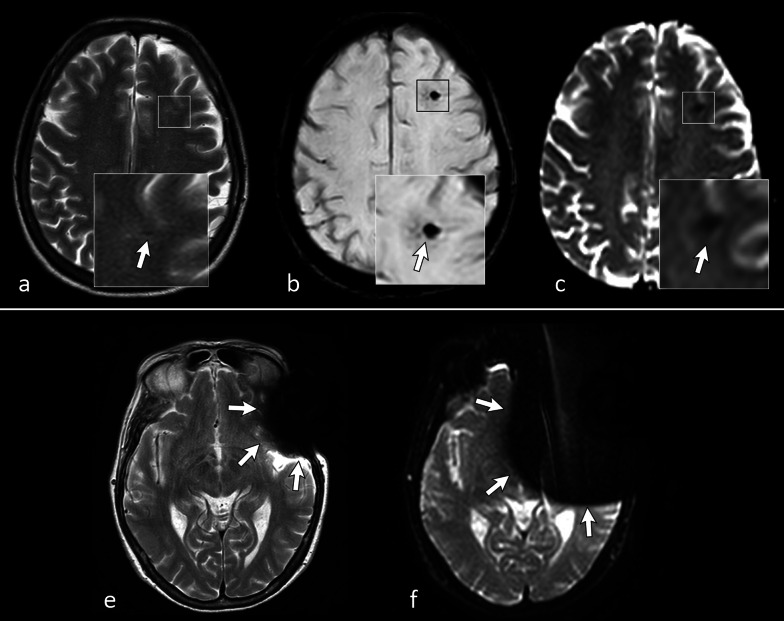
45-year-old woman with an episode of seizure. On axial turbo spin echo (TSE) T2-w. image **a** two almost imperceptible areas of linear hypointensity can be noted (arrow shows the most prominent in the parietal lobe). Axial susceptibility weighted imaging (SWI) scan at the same level (**b**) shows evident susceptibility artifacts caused by hemosiderin within small vascular malformations. The same artifacts can also be noted in echo-planar imaging (EPI) diffusion-weighted image with a b-value of zero (**c**). EPI sequence sensitivity to susceptibility artifact is well depicted by comparison between TSE T2-w. **e** and EPI image (**f**) of the brain in a patient with a ferromagnetic dental device

Echo-planar sequences are the basis for advanced MRI applications such as diffusion, perfusion and functional imaging. In EPI, the use of a long gradient echo train causes images to be more weighted on T2* and to be more dependent on susceptibility effects; this is usually a major drawback for the interpretation of images, but can sometimes be useful as a substitute of GE T2*-w. sequences to detect susceptibility effect not visible on FLAIR or TSE sequences (Fig. 16).

### Metallic artifact reduction sequences (MARS)

With the definition of metallic artifact reduction sequences (MARS) are generally indicated all the MRI techniques which aim to reduce the size and the intensity of artifacts due to magnetic field distortion [84, 85]. Iron, nickel, stainless steel and cobalt—all metals commonly used for prostheses—are ferromagnetic and generate severe artifacts on MRI [15], which are characterized by shape distortion of the metallic object and loss of MRI signal (black artifacts). In addition, rims of high signal intensity (white artifacts) appear around the metallic object. By adopting some expedients based on MRI physics, it is possible to create performing MARS. The increase in the value of turbo factor linearly reduces artifacts, but at the cost of a decrease of the signal-to-noise ratio (SNR). However, the reduction in TE makes it possible to obtain both a reduction of artifacts and an increase in SNR with the only drawback of a slight reduction of the weighting in T2, which does not generally alter image quality. Eventually, reducing slice thickness and increasing matrix size allows a further decrease in artifacts at the cost of a linear reduction of SNR, which can be corrected with more acquisitions of the MR signal. A balanced use of these changes allows to significantly reduce artifacts, without an increase in the acquisition time [86, 87]

In this context, the usage of spectral presaturation with inversion recovery (SPIR) or spectral attenuated inversion recovery (SPAIR) sequences, in order to obtain a selective T2-w. or PD-w. fat-suppression, is not recommended, because the susceptibility due to prostheses impairs this type of fat-suppression. On the other hand, fast-STIR images can provide fat-suppressed images with a good image quality in patients with metal prostheses (Fig. 11).

## Conclusions

When an external magnetic field is applied, all substances (both endogenous and exogenous) are influenced by, and in turn influence, the magnetic field itself. A measure of the magnitude of magnetization of the substance is provided by magnetic susceptibility.

Diamagnetic substances, which comprise most of biological tissues, tend to be repelled by the magnetic field. An example of diamagnetic susceptibility interactions at molecular level is offered by chemical shift imaging, which, exploiting minimal variations in precession frequency of protons belonging to different molecules, allows to explore the relative ratio between water and fat content within a voxel, thus giving valid support in the diagnosis and characterization of common and uncommon pathologies in MRI in both a qualitative and quantitative way.

Paramagnetic, superparamagnetic and ferromagnetic substances include a wide range of endogenous and exogenous materials which are attracted by the applied magnetic field. Knowledge of these substances, of their magnetic properties and of the effect of these properties on the most commonly used sequences in MRI can help practitioners leverage at best the different sequences in order to identify these substances (even when they are present in minimal quantities), to differentiate them when possible and to enhance or reduce the artifacts they create depending on the clinical scenario.

## Data Availability

Not applicable.

## Abbreviations

EPI: Echo-planar imaging
FID: Free induction decay
FLAIR: Fluid attenuated inversion recovery
GE: Gradient echo
IR: Inversion recovery
MARS: Metallic artifact reduction sequences
MRI: Magnetic resonance imaging
NMR: Nuclear magnetic resonance
PD: Proton density
RF: Radiofrequency
SE: Spin echo
SNR: Signal-to-noise ratio
STIR: Short inversion time inversion recovery
SWI: Susceptibility weighted imaging
TE: Echo time
TI: Inversion time
TSE: Turbo spin echo
